# RECRUITING AND RETAINING NURSES AND FRONTLINE CARE WORKERS IN LONG-TERM CARE: THE REACH REALIST REVIEW

**DOI:** 10.1093/geroni/igad104.0775

**Published:** 2023-12-21

**Authors:** Reena Devi, Kirsty Haunch, Sonia Dalkin, Natalie King, Angela Bate, Karen Spilsbury, Claire Goodman, Liz Jones

**Affiliations:** University of Leeds, Leeds, England, United Kingdom; University of Leeds, Leeds, England, United Kingdom; Northumbria University Newcastle, Newcastle, England, United Kingdom; University of Leeds, Leeds, England, United Kingdom; Northumbria University Newcastle, Newcastle, England, United Kingdom; University of Leeds, Leeds, England, United Kingdom; University of Hertfordshire, Hertfordshire, England, United Kingdom; National Care Forum, Nottingham, England, United Kingdom

## Abstract

**Introduction:**

increasing demand for care, high staff turnover and low numbers of new staff are causing significant staff shortages in the long-term-care (LTC) sector. Solutions need to 1) take the heterogeneity of organisational and staff characteristics into account, and 2) make use of the large volume of literature describing the reasons staff join and stay in LTC work. Our research aimed to develop strategies which attract, recruit and retain Registered Nurses and frontline care workers in LTC; explain how and why strategies are effective, for which staff, the conditions needed, and the costs involved.

**Method:**

This realist review involved (i) co-developing strategies and initial programme theories, (ii) systematically searching the evidence to test IPTs and develop theoretical propositions, and (iii) validating and refining propositions with stakeholder groups.

**Results:**

IPTs around the following strategies were rated as important for attracting, recruiting and retaining staff in LTC, and taken forward to the testing phase of our review: rewarding and recognising staff, offering flexible working, career development opportunities, enhanced salary package, welcoming and inducting staff, listening to and providing staff feedback, developing caring work environments and effective staff interviewing. Leadership, caring cultures, teamwork, resources and adequate staffing levels are important conditions which enable these strategies lead to positive outcomes.

**Discussion:**

this is the first study to use a realist approach to developing strategies focused on attracting, recruiting and retaining the LTC workforce. Our findings will be relevant at an international level. Acknowledgement: we thank Karen Winterburn and Edna Feenan for their important input throughout the project. They have both provided care home family perspectives, and have been involved throughout the development and execution of the project.

